# A Scopus-Based Bibliometric Analysis of Global Research Contributions on Milk Fluoridation

**DOI:** 10.3390/ijerph19148233

**Published:** 2022-07-06

**Authors:** Kehinde Kazeem Kanmodi, Jacob Njideka Nwafor, Afeez Abolarinwa Salami, Eyinade Adeduntan Egbedina, Lawrence Achilles Nnyanzi, Temitope Oluwabukola Ojo, Ralph M. Duckworth, Fatemeh Vida Zohoori

**Affiliations:** 1 School of Health and Life Sciences, Teesside University, Middlesbrough TS1 3BX, UK; b1148611@tees.ac.uk (K.K.K.); b1210723@live.tees.ac.uk (E.A.E.); l.nnyanzi@tees.ac.uk (L.A.N.); b1148783@live.tees.ac.uk (T.O.O.); ralphduckworth@talktalk.net (R.M.D.); 2 Cephas Health Research Initiative Inc., Ibadan 21605, Nigeria; jacob.nwafor@nuh.nhs.uk (J.N.N.); donaphice89@gmail.com (A.A.S.); 3 Department of Medicine, Nottingham University Hospital NHS Trust, Nottingham NG2 4LA, UK; 4 Department of Oral and Maxillofacial Surgery, University College Hospital, Ibadan 200285, Nigeria

**Keywords:** milk, fluoride, fluoridation, bibliometric, analysis

## Abstract

Fluoridated-milk schemes have been developed and implemented in many countries to prevent dental caries. This study aimed to evaluate the impact/influence of scientific publications, researchers, and institutions conducting research on milk fluoridation; to explore the international and inter-institutional collaboration and illustrate scientific output trends; and to pinpoint research hotspots in milk fluoridation research. This bibliometric analysis of original research articles on milk fluoridation includes all of the original articles published in peer-reviewed journals systematically extracted from the SCOPUS database. In total, 108 articles were included in this study, with a total of 11,789 citations. A majority (67.6%) of these articles were in the subject area of ‘dentistry’, 22.2% externally funded, 14.8% published in the journal, Caries Research, 7.4% authored/co-authored by Twetman S, 6.5% by authors from Universidad de Chile, and the UK had the highest output (24.1%). The network visualizations showed that those countries with current/past histories of implemented milk fluoridation programs were interconnected on the network visualization map, and they were predominantly the hotspots for original research on milk fluoridation. This study also identified inequalities in research outputs on the topic. With the current enormous global burden of dental caries in children, particularly in low- and middle-income countries, there is an urgent need for greater and more equitable funding of milk fluoridation research globally.

## 1. Introduction

Dental caries is a global health problem, affecting all socioeconomic strata [1]. Pertinently, about 60 to 90% of schoolchildren have experienced dental caries [2]. The global health impact of dental caries is enormous–it affects the quality of life, growth, and the development of children, leaving significant psychological impacts on both the children and their families [3,4,5].

Dental caries is a chronic multifactorial infectious disease of bacterial origin, that causes the demineralization of the inorganic components of the tooth as a result of acid production on the tooth surface from fermentable carbohydrates, thereby leading to cavity formation [6,7,8].

Fluoride has been identified as a protective factor in dental caries control, as it promotes re-mineralization and inhibits the demineralization of the enamel [9,10]. The re-mineralization involves the deposition of calcium phosphates from the saliva to rebuild the partly dissolved enamel crystals [10]. Although the main action of fluoride for caries control is primarily topical (i.e., the presence of low concentrations in the oral fluids), evidence from cohort studies also supports its systemic effects (i.e., fluoride’s incorporation into the apatite crystals of developing teeth, making them more resistant to acid demineralization) [11]. In view of this, multiple preventive public health fluoridation programs including the fluoridation of water, salt, and milk have been developed and implemented in several parts of the world [12,13,14,15]. These programs have been evaluated and were found to be successful, reducing the need to rely on treatment-based programs, especially in places where the access to oral care is inadequate [12,13,14,15].

Milk remains an important part of the human diet as a good source of protein, minerals (e.g., calcium), trace elements (e.g., zinc), and fat-soluble vitamins. As a nutritious food, it is offered to school-aged children in many countries as part of child nutrition programs in deprived areas [15,16,17,18,19]. Due to its non-cariogenicity, milk has been suggested as a vehicle for delivering fluoride to school children in non-fluoridated water areas [18,19]. Fluoridated milk is also relatively economical and easy to produce [15,18,19].

Since the introduction of milk fluoridation in the 1950s [20], various in vitro, in vivo, and epidemiological studies have been conducted on its oral health effects. Although multiple narrative and systematic reviews have been published on milk fluoridation [15,21,22,23,24], no study has bibliometrically analyzed the global research contributions on this topic.

Bibliometric analysis is a rigorous research method, which aims to statistically measure the impact/influence of scientific publications, researchers, and institutions conducting research on a research topic [25,26,27,28,29,30,31]. It has gained huge popularity in recent years, due to the advancement and availability of bibliometric software, such as VOSviewer (Centre for Science and Technology Studies, Leiden University, Leiden, The Netherlands), and scientific databases, such as Scopus [32]. Within recent decades, researchers have used bibliometric analysis to evaluate international and inter-institutional collaborations, to illustrate scientific output trends of a specific research topic, and to pinpoint research hotspots [33]. However, despite the availability of authoritative guides in systematic reviews, they do not offer a satisfactory breadth and depth on the bibliometric methodology [32]. Regardless of its limitations, bibliometric analysis is regarded as a scientific method to summarize and synthesize the published literature on a specific topic [32].

Although there are several reviews, including systematic reviews, on fluoridated milk [34], a bibliometric analysis of milk fluoridation is lacking. Therefore, the aim of this paper was to conduct such an analysis of the global research output on milk fluoridation, from inception until now. The objectives were to evaluate the impact/influence of the scientific publications, researchers, and institutions conducting research on milk fluoridation, as such studies explored the international and inter-institutional collaborations, illustrated the scientific output trends, and pinpointed the research hotspots in milk fluoridation research.

## 2. Materials and Methods

### 2.1. Study Type

This study was a bibliometric analysis of original research articles published on milk fluoridation, from inception until 28 March 2022.

### 2.2. Data Source

The study data were obtained from the SCOPUS electronic database. This database was used here owing to its global recognition in terms of credibility, comprehensiveness, prestige, suitability, and popularity in bibliometric analyses [35,36,37,38]. A combination of the search terms, including “fluori*” AND “milk” AND (“fluoridation” OR “fluoridated”) were used to retrieve highly relevant publications on milk fluoridation from SCOPUS. The search yielded a total of 357 publications.

### 2.3. Data Extraction

Selection criteria (inclusion and exclusion) were developed, as shown in Table 1.

The titles and abstracts of the retrieved articles were then screened by three independent reviewers (KKK, EAE, and TOO), based on the selection criteria. In cases where there was conflict in the selection of an original article, another reviewer (FVZ) resolved the conflict.

The following information was extracted: data concerning citation information (authors’ names and identity, document title, year of publication, journal title, volume, issue and page numbers, and citation count); bibliographical information (affiliations, serial identifiers of journal, language of original document, and journal publisher); author keywords; and funding details were exported into a “.csv” (comma separated values) format for analysis.

### 2.4. Data Analysis

Microsoft Office Excel 2021 (Microsoft Corporation, Washington, DC, USA) was used to analyze the bibliometric parameters at the level of publications (e.g., year of publication, number of citations, highly cited articles), author (most prolific and most highly cited author, institute, country), as well as at the level of social structure (e.g., co-authorship network), and of conceptual structure (e.g., co-occurrence network for keywords).

The VOSviewer (Centre for Science and Technology Studies, Leiden University, Leiden, The Netherlands) was used to create several network visualizations including keyword co-occurrence, co-authorships (covering authors, institutions, countries/territories), and citations (covering journals, authors, institutions, countries/territories).

In the creation of network visualizations, the threshold for inclusion of an entity was having at least: (i) two occurrences of a key word for keyword co-occurrence; (ii) two articles; and one citation for co-authorships; and (iii) one article; and five citations for citations.

A network visualization is made up of items, clusters, and links [39]. An item refers to an author/institution/country/territory/keyword. A cluster refers to a set of items included in a network visualization map [39]. A link is an association between two items. Furthermore, the link between two items has its strength (total link strength (TLS)) which is a positive numerical value [39]. A TLS represents the total number of characteristics (e.g., total citations, total co-authorships, and total co-occurrences) that an item has in common with other items [39]. For example, in the network visualization of the co-occurrence of keywords, a TLS of 10 indicates that such a keyword co-occurred with 10 other keywords.

Furthermore, in the network visualizations, each cluster has its own color and the cluster with the highest TLS appears in red. In addition, the bigger the font size of an item, the greater the weight of the item. The weight of all of the items analyzed in all of the visualizations was the total number of articles related to such an item.

It should be noted that when generating network visualizations in VOSviewer, not all of the entities included in each network visualization were depicted in the visualizations/figures, as some names/entities were hidden due to the overlap of items in the network [39].

## 3. Results

### 3.1. Description of the Included Studies

**Figure 1:**
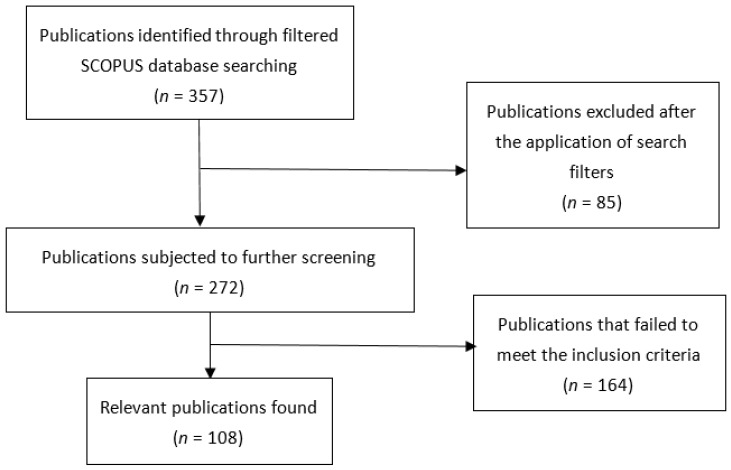
Flow chart of the screening process.

In total, 357 publications were retrieved from the basic search, of which 85 were non-original research articles and were therefore excluded. Of the remaining 272 original research articles, 108 met the inclusion criteria and are included in this bibliometric analysis (Figure 1). The full list of included articles is in File S1 (Supplementary Materials).

### 3.2. Publication Trends

**Figure 2:**
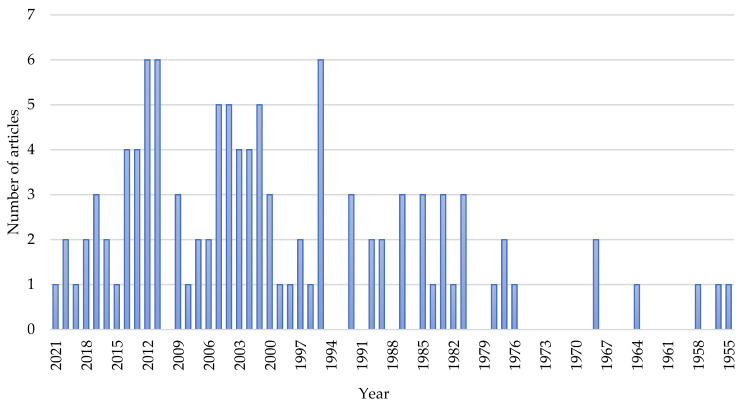
Publication trend of original research articles on milk fluoridation.

Figure 2 shows the publication-year trend of original research articles on milk fluoridation. The first article was published in 1955, and the years with the highest frequency of articles were 1995, 2011, and 2012. Over the decades (1955–2020), the output rates of these articles have increased (1955–1980 = 10; 1981–1990 = 18; 1991–2000 = 17; 2001–2010 = 31; 2011–2020 = 31).

### 3.3. Citation Trend

**Figure 3:**
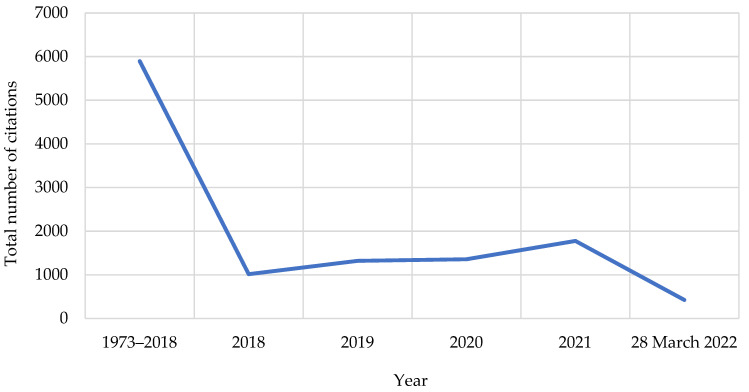
Citations of original research articles on milk fluoridation by year.

The 108 included original articles were cited in a further 609 documents (original research articles, reviews, books, etc.) with a total of 11789 citations (Figure 3) and an h-index of 52. The article with the first citation ever was published in 1973 in German–“Büttner, W. et al., 1973” [40].

### 3.4. Subject Areas

**Figure 4:**
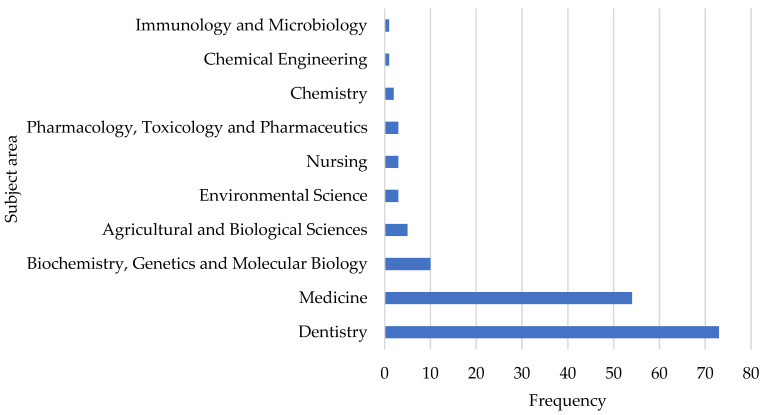
Distribution of original research articles by subject area.

The majority (67.6%) of the original research articles published on milk fluoridation were in the subject area of ‘dentistry’, while only one article (0.9%) each was in ‘chemical engineering’ and ‘immunology and microbiology’ (Figure 4).

### 3.5. Research Funders

**Figure 5:**
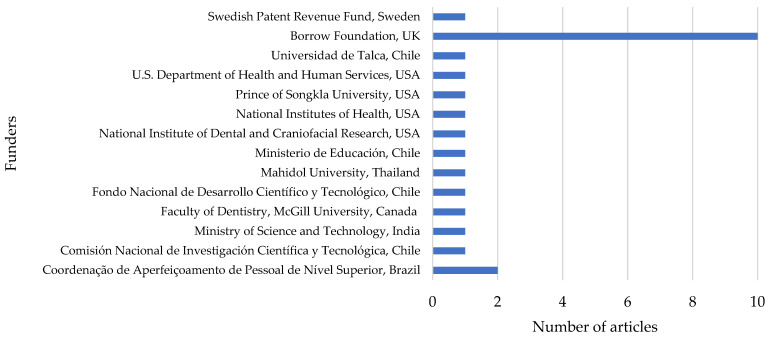
Funders of original research on milk fluoridation.

Out of the 108 articles on milk fluoridation, only 24 (22.2%) were externally funded. Figure 5 shows the names of all of the funding organizations (*n* = 14). Out of these, three were in the USA (National Institutes of Health, National Institute of Dental and Craniofacial Research, and U.S. Department of Health and Human Services).

All of the funding organizations funded one original research article, except for Coordenação de Aperfeiçoamento de Pessoal de Nível Superior (Brazil) and the Borrow Foundation (UK), which funded two and ten articles, respectively.

### 3.6. Top Ten Journals

All of the included original research articles were published in 50 peer-reviewed journals. The top ten journals, together with the corresponding CiteScore 2020 and frequencies of such articles, are listed in Table 2. CiteScore 2020 is a ranking score used by SCOPUS; it is calculated as follows [41]:

(1) $$\mathrm{CiteScore}\;2020=\frac{\begin{array}{c} \mathrm{Number}\;\mathrm{of}\;\mathrm{ciations}\;\mathrm{received}\;\mathrm{in}\;2017-2020\;\mathrm{to}5\\ \mathrm{published}\;\mathrm{document}\;\mathrm{types}\;(\mathrm{articles},\;\mathrm{etc}.)\;\mathrm{by}\;a\;\mathrm{journal}\;\mathrm{in}\;\mathrm{the}\\ \mathrm{same}\;4\;\mathrm{years} \end{array}}{\begin{array}{c} \mathrm{Total}\;\mathrm{number}\;\mathrm{of}\;\mathrm{documents}\;\mathrm{indexed}\;\mathrm{in}\;\mathrm{SCOPUS}\;\mathrm{and}\\ \mathrm{published}\;\mathrm{in}\;2017-2020 \end{array}}$$

Caries Research was the journal with the highest frequency of such articles, while the Journal of Dental Research was the journal with the highest CiteScore 2020.

### 3.7. Top Ten Authors

A total of 271 authors wrote the included original articles on milk fluoridation, of whom the top ten are listed in Table 3. Twetman S, with a current/most recent affiliation with Københavns Universitet (Copenhagen, Denmark), had the highest number (*n* = 8) of such articles.

### 3.8. Top Ten Institutions

A total of 183 institutions sourced the included original articles on milk fluoridation. Table 4 shows the list of the top ten institutions having the highest number of these articles. Half of these institutions were UK-based. The Universidad de Chile (Chile), University of Liverpool (UK), Általános Orvostudományi Kar (Hungary), and Umeå Universitet (Sweden) were the institutions with the highest number (*n* = 7, each) of such articles.

### 3.9. Top Ten Countries

A total of 26 countries were identified as the country affiliations of the authors of the original articles on milk fluoridation: Table 5 lists the top ten countries. Half of these countries were European. Furthermore, UK (*n* = 26), United States of America (*n* = 14), and Sweden (*n* = 10) were the top three countries on the list.

### 3.10. Top Ten Articles

Table 6 lists the top ten original research articles on milk fluoridation, based on citations. These articles were published between 1984 and 2014, and their citations range from 24 to 114. The article with the highest number of citations was published in 2009, in Caries Research.

### 3.11. Network Visualization of Co-Occurrence

**Figure 6:**
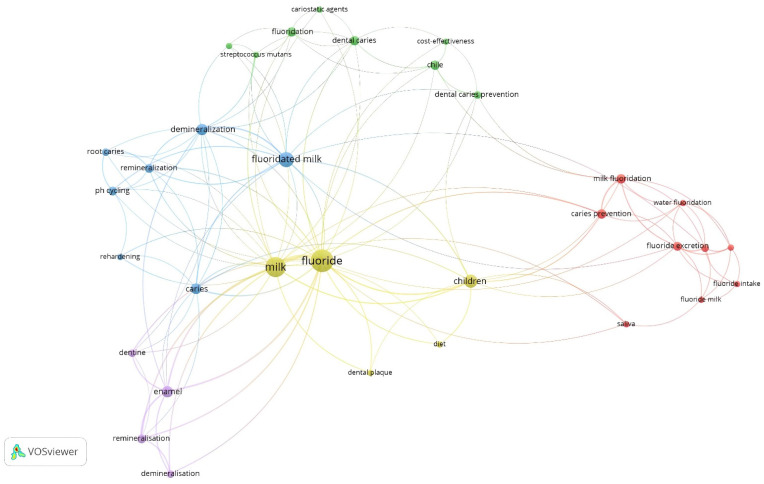
Network visualization of co-occurrence of author keywords.

An analysis of the authors’ keywords was used, to explore co-occurrence. Only those keywords used by the authors of the included original research articles on milk fluoridation, and which appeared at least twice, are illustrated in Figure 6. Of the 128 author keywords, only 33 met the threshold (i.e., appeared in at least two articles). The three most frequently appearing keywords were “fluoride” (total link strength (TLS) = 70; occurrence rate = 26 articles), “milk” (TLS = 58; occurrence rate = 21 articles), and “fluoridated milk” (TLS = 24; occurrence rate = 12 articles).

It is also noteworthy that all of the keywords in the network visualization are linked, and none was isolated. All of the clusters, except one, contain at least one fluoride-specific term.

### 3.12. Network Visualization of Co-Authorship

A total of 271 authors, 183 institutions, and 26 countries/territories were involved with the included original articles on milk fluoridation. For the network visualizations of co-authorship, all of the items (authors, institutions, and countries/territories) meeting the threshold were included, regardless of interconnectivity—this was undertaken to holistically identify the global pattern of research collaboration among these items of interest.

#### 3.12.1. Authors

**Figure 7:**
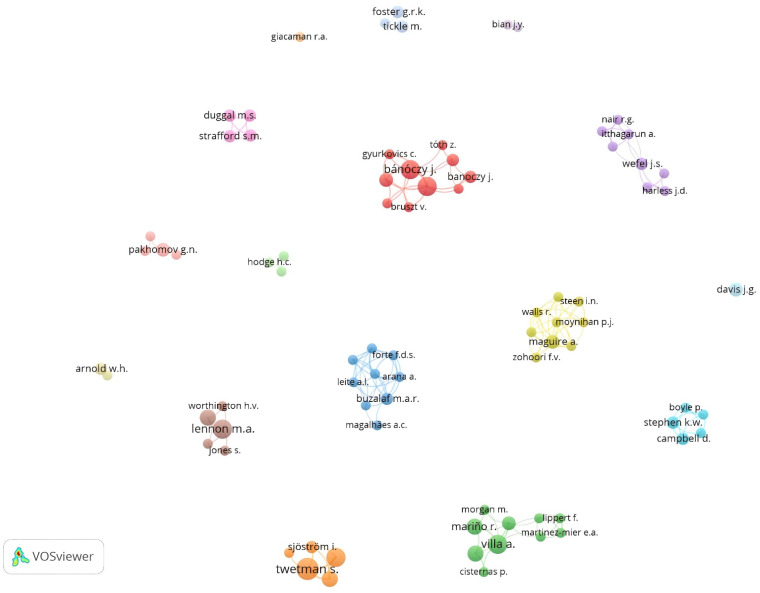
Network visualization of co-authorship among authors.

In the analysis of authors, only 81 authors, occurring in 16 clusters, met the threshold (Figure 7). The cluster with the highest TLS has ten authors, with Zimmermann P. as the author with the highest TLS (TLS = 20; total citations (TC) = 64). In addition, Zimmermann P. had the highest TLS amongst the 81 authors in the network visualization.

#### 3.12.2. Institutions

Only nine institutions, occurring in seven clusters, met the threshold:

Cluster 1: University of New England (TLS = 4, TC = 39) and University of Chile (TLS = 3, TC = 35); Cluster 2: Federal University of Paraiba (TLS = 2, TC = 41) and Peruvian University (TLS = 2, TC = 3); Cluster 3: Indiana University (TLS = 0, TC = 21); Cluster 4: University of Liverpool (TLS = 0, TC = 33); Cluster 5: University of Iowa (TLS = 0, TC = 24); Cluster 6: University of Manchester (TLS = 2, TC = 3); Cluster 7: Teesside University (TLS = 0, TC = 13).

Furthermore, none of the clusters were interconnected in the visualization.

#### 3.12.3. Countries/Territories

Only 17 countries, occurring in 8 clusters, met the threshold:

Cluster 1: Australia (TLS = 7, TC = 7), Chile (TLS = 7, TC = 105) and Thailand (TLS = 1, TC = 8); Cluster 2: Bulgaria (TLS = 1, TC = 8), Hungary (TLS = 2, TC = 86), and United Kingdom (TLS = 5, TC = 237); Cluster 3: China (TLS = 1, TC = 30) and Hong Kong (TLS = 3, TC = 38); Cluster 4: Czech Republic (TLS = 2, TC = 24) and United States (TLS = 7, TC = 187); Cluster 5: Germany (TLS = 1, TC = 66) and Switzerland (TLS = 3, TC = 59); Cluster 6: Brazil (TLS = 5, TC = 81) and Peru (TLS = 2, TC = 41); Cluster 7: Denmark (TLS = 5, TC = 147) and Sweden (TLS = 4, TC = 186); Cluster 8: New Zealand (TLS = 0, TC = 5)

All of the clusters were interconnected, except for Cluster 8 which was isolated from the others.

### 3.13. Network Visualization of Citations

A total of 50 journals published the included original articles on milk fluoridation. The network visualization of the citations was based on the journals, authors, institutions, and countries. Only the interconnected entities (i.e., journals, authors, institutions, and countries/territories), meeting the threshold, were included in the network visualization.

#### 3.13.1. Journals

**Figure 8:**
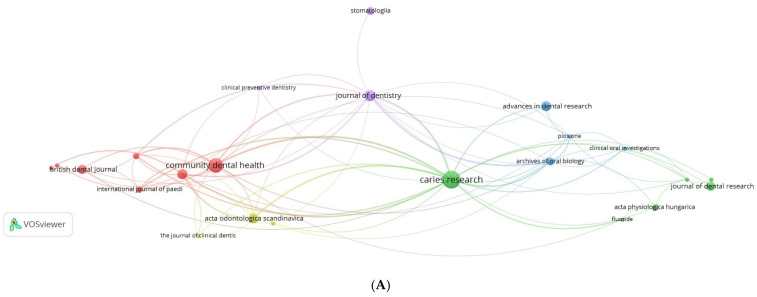

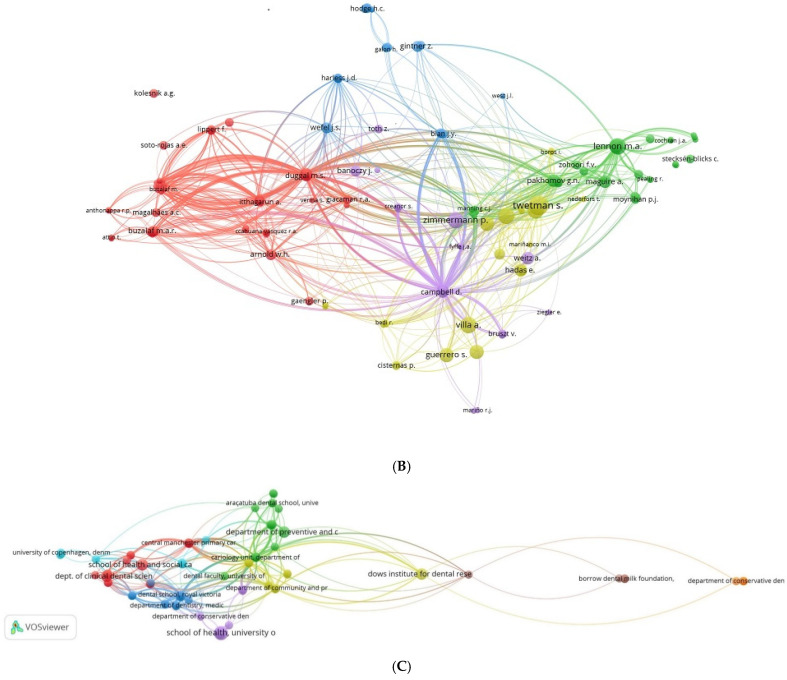

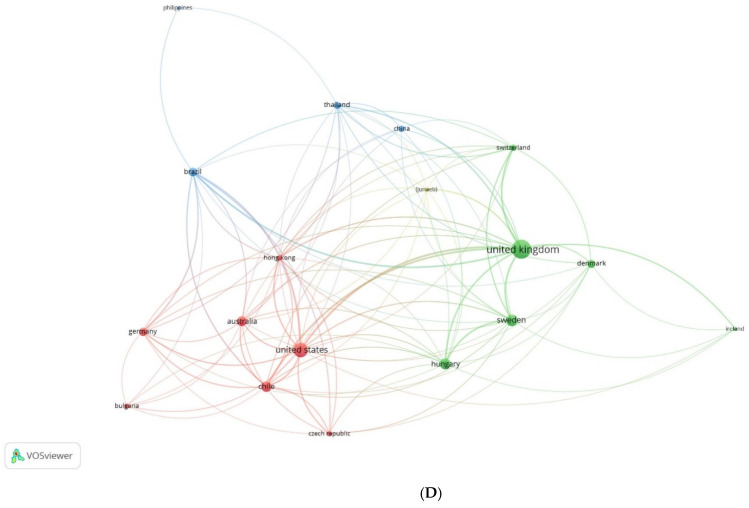
(**A**) Network visualization of citations among journals; (**B**) Network visualization of citations among authors; (**C**) Network visualization of citations among institutions; (**D**) Network visualization of citations among countries and territories.

Out of the 26 journals meeting the threshold, only 23 of the journals were interconnected, occurring in 5 clusters (Figure 8A). The cluster with the highest TLS included seven journals. Caries Research was the journal with the highest TLS (TLS = 76; TC = 299) in the visualization.

All of the journals were interconnected to at least two journals, except for four journals which were connected to one journal or none: Stomatologiya (TLS = 1, TC = 7), American Journal of Clinical Nutrition (TLS = 0, TC = 41), ASDC Journal of Dentistry for Children (TLS = 0, TC = 10), and BMC Oral Health (TLS = 0, TC = 7). The only interconnected journal to Stomatologiya was the Journal of Dentistry (TLS = 42, TC = 79).

#### 3.13.2. Authors

Only 172 authors met the threshold, of which 147 were interconnected in 5 clusters (Figure 8B). The cluster with the highest TLS included 45 authors. Twetman S was the author with the highest TLS (TLS = 158; TC = 186) in the visualization.

Almost all (*n* = 145) of the authors in the network visualization were interconnected to at least seven authors each, except for: Marino RJ (TLS = 5, TC = 9); Villa AE (TLS = 5, TC = 9); and Kolesnik AG (TLS = 4, TC = 5).

#### 3.13.3. Institutions

Out of the 109 institutions which met the threshold, only 87 were interconnected in 5 clusters (Figure 8C). The cluster with the highest TLS included 17 institutions. The Dows Institute for Dental Research (USA) was the institution with the highest TLS (TLS = 37; TC = 14) in the visualization.

All of the institutions captured in the network visualization, except for ten, were interconnected to at least two institutions each. The following ten institutions were connected to only one institution each: Borrow Foundation (TLS = 1, TC = 13); Simmelweis University (TLS = 1, TC = 13); Leeds University (TLS = 1, TC = 7); Ministry of Welfare–Budapest (TLS = 1, TC = 13); University of Copenhagen (TLS = 1, TC = 7); University of Dundee (TLS = 1, TC = 6); University of Glasgow (TLS = 1, TC = 6); University of Plymouth (TLS = 1, TC = 6); and the World Health Organization–Geneva (TLS = 1, TC = 7).

#### 3.13.4. Countries/Territories

Only 21 countries and territories met the analytic threshold, of which 18 were interconnected, occurring in 4 clusters (Figure 8D). The cluster with the highest TLS included seven countries/territories. The UK was the country with the highest TLS (TLS = 100; TC = 237) in the visualization.

All of the countries and territories captured in the network visualization, except for two, were interconnected to at least ten countries/territories each. These two countries were Bulgaria (TLS = 8, TC = 8) and the Philippines (TLS = 3, TC = 6).

## 4. Discussion

The findings obtained in this study are noteworthy. Overall, the total global original research output on milk fluoridation is low. However, as shown in Figure 2 and Figure 3, research interest in milk fluoridation has grown over the past seven decades (1950s–2020), as the topic had, since inception till date, garnered citations and outputs on a yearly basis. This shows that the idea of milk fluoridation has received increasing attention, from its inception until today. However, a focus on the past two decades (2000–2010 versus 2010–2020) shows no obvious increase, as both decades had the same number of publications. This may be because of the shifted interest of many European and American scientific dental associations concerning systemic fluoride use in 2000 to 2020. Most of these associations became skeptical about recommending the use of fluoride supplements as a standard approach for the prevention of dental caries, due to its associated risk of dental fluorosis [41,52,53]. Although the idea of fluoridation was initially conceived in the USA, the present findings indicate that the UK is currently leading in terms of original research output on milk fluoridation (Table 5). This may be because the UK is the location of the top funder of milk fluoridation research (the Borrow Foundation) in the world, as evidenced by our study findings (Figure 5). Despite the significant research output on milk fluoridation in the UK, this has not been translated into a widespread milk fluoridation-based caries prevention program in the UK itself, as such programs are only being implemented in selected areas characterized by high levels of dental caries [54].

Milk fluoridation programs have been found to be effective in dental caries prevention across populations [18,41,52,53]. However, only a few countries have ever implemented such programs; these countries include Bulgaria, Chile, UK, Russia, Thailand, Brazil, Peru, Hungary, Israel, USA, and others [18,41,52,53]. This may explain why most of the countries/institutions with the highest original research outputs on milk fluoridation, as obtained in the present study (Table 4 and Table 5), have a history of past/current implementation of milk fluoridation programs.

To the best of the authors’ knowledge, no African country is currently implementing a community-based milk fluoridation program. Unlike in many European countries where milk is a relatively cheap and common staple food, milk is less common in Africa and is relatively expensive [55,56]. In addition, due to the relatively hot climate of Africa, milk preservation requires a lot of resources (e.g., constant electricity, access to a fridge, etc.) which are limited in Africa [57,58,59]. These factors may jointly explain why no African country or institution made the top ten countries/institutions with original research contributions on milk fluoridation. Furthermore, this could be attributable to an inadequate human research capacity and a lack of infrastructure for conducting research on milk fluoridation on the African continent. This highlights the need for increased research resources on this topic in Africa. By so doing, this will enable African scientists to contribute to the development of this topic in the African setting, and this will also contribute towards the dental caries prevention efforts there. Dental caries is a public health issue in Africa; unfortunately, the adoption of modern oral hygiene practices in many African countries is yet to catch up with practices in more developed countries [60].

In terms of citation, the most cited article (Table 3 and Table 6) was a randomized controlled trial published in 2009 by Stecksén-Blicks et al. [42]. In the hierarchy of scientific evidence, systematic reviews are placed highest, while randomized control trials are placed second highest [61]. However, amongst the original research papers, randomized control trials are placed highest, which may explain why the article by Stecksén-Blicks et al. [42], being a randomized control trial, was the most cited original research article on milk fluoridation. Many scientists will have referred to the article for evidence.

The findings obtained from the network analyses were noteworthy as well. Regarding co-occurrence, “milk” and “fluoride”, as evident in Figure 6, were the two most used keywords in the original research articles on milk fluoridation. The other keywords were children, demineralization, and remineralization. The reason for this high usage rate is not surprising—these articles were written on the topic of milk fluoridation and prevention of dental caries, mainly in children.

At the author level, Zimmermann P—a researcher from Hungary—was the author with the most prolific collaboration network on milk fluoridation research globally (Figure 7). At the institutional level, the cluster/group, comprising of the University of New England (Australia) and the University of Chile (Chile), was the most prolific research collaboration on milk fluoridation research globally. At the country level, Australia, Chile, and Thailand had the highest volume of co-sourced articles on milk fluoridation. Overall, this shows that most of the research outputs on milk fluoridation were related to the countries with past/current histories of implementation of milk fluoridation programs [15]. In addition, similar observations were made concerning the citation patterns of these outputs (Figure 8A–D).

Furthermore, we observed that the multiple clusters/groups of researchers/institutions/countries/territories authoring or sourcing articles on milk fluoridation were very small in size, with little or no collaboration between the groups. The different factors, including language barriers and limited funding, may be responsible for the current lack of large collaborations in milk fluoridation research [62]. However, due to the limited scope of this review, the specific factors responsible for this observation could not be ascertained. Therefore, we recommend the need for further studies to identify these factors. The existing research outputs on milk fluoridation span multiple academic disciplines (Figure 4), with most of them classified under the “dentistry” subject area. However, none of these outputs were classifiable under the “economics, econometrics, and finance” and “business, management, and accounting” subject areas. This shows that researchers are yet to substantially explore the monetary and economic aspects of milk fluoridation. Therefore, there is a need for research in these areas.

Only 22.2% of the original research articles on milk fluoridation were externally funded, which shows that the funding on this topic is low. Due to the high relevance and effectiveness of fluoridated milk as a dietary component and as a vehicle for fluoride delivery [24], the need for research funding, particularly in African countries, on milk fluoridation cannot be over-emphasized. Therefore, it is recommended that global health funding agencies should focus more on this research topic.

This study has limitations. Only the original research articles indexed in the SCOPUS database were included in this study; hence, articles only indexed elsewhere were excluded. Therefore, the list of original research articles on milk fluoridation obtained in this study may not be all-inclusive. Notwithstanding this limitation, this study is believed to be the first study to bibliometrically analyze the original research output on milk fluoridation. In addition, the findings obtained here have provided deep insights regarding the global milk fluoridation research landscape.

## 5. Conclusions

This study shows an increasing global research interest (as measured by citations, outputs, etc.) in milk fluoridation. It has also identified inequalities in research on the topic; for example, there are no outputs from any African country. The UK is the country with the highest output of original research on the topic, and this may be due to the significant funding from the Borrow Foundation. With the current enormous global burden of dental caries in children, there is an urgent need for more, funding of milk fluoridation research globally.

## Figures and Tables

**Table 1:** Selection criteria.

Inclusion Criteria	Exclusion Criteria
Original research articles	Books, book chapters, editorials, commentaries, review articles, letters to the editor, and other publications that are not original research articles
Articles that were highly relevant to fluoridated milk and milk fluoridation (this is determined by the mentioning of fluoridated milk or milk fluoridation in the title and/or abstract)	Irrelevant articles (this refers to articles that did not mention fluoridated milk or milk fluoridation in the title and/or abstract)
Articles where their title and abstract were translated into English	Articles that lacked English translation

**Table 2:** Top 10 journals where original research articles on milk fluoridation were published.

Journal Title	CiteScore 2020	Frequency
Caries Research	5.2	16
Community Dental Health	1.5	11
Journal of Dentistry	6.2	6
Acta Odontologica Scandinavica	2.8	5
Advances in Dental Research	8.2	5
Community Dentistry and Oral Epidemiology	4.4	5
British Dental Journal	1.4	4
Journal of Dental Research	9.9	4
Archives of Oral Biology	3.9	3
Stomatologiya	0.3	3

**Table 3:** Top ten authors of original research articles on milk fluoridation.

Authors	Current/Most Recent Affiliation on SCOPUS) *	Total Outputs
Twetman, S.	Københavns Universitet, Copenhagen, Denmark	8
Banoczy, J.	Általános Orvostudományi Kar, Budapest, Hungary	7
Lennon, M.A.	The University of Sheffield, Sheffield, United Kingdom	7
Petersson, L.G.	Halland Hospital, Halmstad, Sweden	7
Villa, A.	University de Chile, Santiago, Chile	7
Zimmermann, P.	Általános Orvostudományi Kar, Budapest, Hungary	7
Engstrom, K.	Umeå Universitet, Umeå, Sweden	5
Guerrero, S.	Universidad de Chile, Santiago, Chile	5
Ketley, C.E.	University of Liverpool, Liverpool, United Kingdom	5
Marino, R.	University of Melbourne, Parkville, Australia	5

* The current/most recent affiliation of these authors were obtained from SCOPUS.

**Table 4:** Top ten institutions having highest number of original research articles on milk fluoridation.

Institution	Country	Total Output
Universidad de Chile	Chile	7
University of Liverpool	UK	7
Általános Orvostudományi Kar	Hungary	7
Umeå Universitet	Sweden	7
Newcastle University	UK	5
University of Manchester	UK	5
Uniseridade de São Paulo	Brazil	4
Leeds Dental Institute	UK	4
Teesside University	UK	4
Københavns Universitet	Denmark	4

**Table 5:** Top ten countries with the highest number of original research articles on milk fluoridation.

Country	Continent	Total Output
United Kingdom (UK)	Europe	26
United States of America	North America	14
Sweden	Europe	10
Chile	South America	8
Hungary	Europe	8
Australia	Australia	7
Brazil	South America	6
Denmark	Europe	5
Germany	Europe	5
Thailand	Asia	4

**Table 6:** Top ten original research articles on milk fluoridation, and total citations.

Articles	Number of Citations (SCOPUS)
Stecksén-Blicks et al., 2009 [42]	114
Ophaug et al., 1985 [43]	47
Stephen et al., 1984 [44]	39
Wiegand et al., 2014 [45]	28
Mariño et al., 2012 [46]	28
Mariño et al., 2001 [47]	26
Bánóczy et al., 1985 [48]	26
Pratten et al., 2000 [49]	25
Bian et al., 2003 [50]	24
Ketley et al., 2001 [51]	24

## Data Availability

Not applicable.

## Supplementary Materials

The following supporting information can be downloaded at: https://www.mdpi.com/article/10.3390/ijerph19148233/s1, File S1: Scopus document.

## References

[1] Al Agili D.E. (2013). A systematic review of population-based dental caries studies among children in Saudi Arabia. Saudi Dent. J..

[2] Petersen P.E., Bourgeois D., Ogawa H., Estupinan-Day S., Ndiaye C. (2005). The global burden of oral diseases and risks to oral health. Bull. World Health Organ..

[3] Mathur V.P., Dhillon J.K. (2018). Dental Caries: A Disease Which Needs Attention. Indian J. Pediatr..

[4] Knapp R., Marshman Z., Gilchrist F., Rodd H. (2020). The impact of dental caries and its treatment under general anaesthetic on children and their families. Eur. Arch. Paediatr. Dent..

[5] Gomes M.C., Perazzo M.F., Barbosa Neves É.T., Firmino R.T., Lopes R.T., Assunção C.M., Ferreira F.M., Paiva S.M., Granville-Garcia A.F. (2021). The impact of dental pain due to caries in the oral health-related quality of life of children. J. Dent. Child..

[6] Conrads G., About I. (2018). Pathophysiology of Dental Caries. Monogr. Oral Sci..

[7] Maher R., Khan A., Rahimtoola S., Bratthall D. (1992). Prevalence of mutans streptococci and dental caries in Pakistani children. J. Pak. Med. Assoc..

[8] Bowen W. (2016). Dental caries-not just holes in teeth! A perspective. Mol. Oral. Microbiol..

[9] Carey C.M. (2014). Focus on Fluorides: Update on the Use of Fluoride for the Prevention of Dental Caries. J. Evid. Based Dent. Pract..

[10] Cate J.M.T. (2013). Contemporary perspective on the use of fluoride products in caries prevention. Br. Dent. J..

[11] Buzalaf M.A.R., Pessan J.P., Honorio H.M., Ten Cate J.M. (2011). Mechanisms of action of fluoride for caries control. Monogr. Oral Sci..

[12] Whelton H., Spencer A., Do L., Rugg-Gunn A. (2019). Fluoride Revolution and Dental Caries: Evolution of Policies for Global Use. J. Dent. Res..

[13] Marthaler T.M. (2013). Salt fluoridation and oral health. Acta Med. Acad..

[14] Iheozor-Ejiofor Z., Worthington H., Walsh T., O’Malley L., Clarkson J.E., Macey R., Alam R., Tugwell P., Welch V., Glenny A.-M. (2015). Water fluoridation for the prevention of dental caries. Cochrane Database Syst. Rev..

[15] Bánóczy J., Rugg-Gunn A., Woodward M. (2013). Milk fluoridation for the prevention of dental caries. Acta Med. Acad..

[16] Mariño R., Morgan M., Weitz A., Villa A. (2007). The cost-effectiveness of adding fluorides to milk-products distributed by the National Food Supplement Programme (PNAC) in rural areas of Chile. Community Dent. Health.

[17] Pakhomov G.N., Ivanova K., Moller I.J., Vrabcheva M. (1995). Dental Caries-reducing Effects of a Milk Fluoridation Project in Bulgaria. J. Public Health Dent..

[18] Bánóczy J., Rugg-Gunn A.J., Bánóczy J., Petersen P.E., Rugg-Gunn A.J. (2009). Clinical studies. Milk Fluoridation for the Prevention of Dental Caries.

[19] Mariño R.J., Villa A.E., Weitz A., Guerrero S. (2004). Caries prevalence in a rural Chilean community after cessation of a powdered milk fluoridation program. J. Public Health Dent..

[20] Ziegler E. (1956). Uber die Milchfluorierung [Fluoridation of milk]. Bull. Schweiz Akad. Med. Wiss..

[21] Yeung C.A. (2008). A systematic review of the efficacy and safety of fluoridation. Evid.-Based Dent..

[22] Cagetti M.G., Campus G., Milia E., Lingström P. (2013). A systematic review on fluoridated food in caries prevention. Acta Odontol. Scand..

[23] Bánóczy J., Rugg-Gunn A.J. (2007). Caries prevention through the fluoridation of milk. A review. Fogorv. Szle..

[24] O’Mullane D.M., Baez R.J., Jones S., Lennon M.A., Petersen P.E., Rugg-Gunn A.J., Whelton H., Whitford G.M. (2016). Fluoride and Oral Health. Community Dent. Health.

[25] Zhang J., Cenci J., Becue V., Koutra S., Ioakimidis C.S. (2020). Recent Evolution of Research on Industrial Heritage in Western Europe and China Based on Bibliometric Analysis. Sustainability.

[26] Ahmad P., Slots J. (2020). A bibliometric analysis of periodontology. Periodontology.

[27] Akmal M., Hasnain N., Rehan A., Iqbal U., Hashmi S., Fatima K., Farooq M.Z., Khosa F., Siddiqi J., Khan M.K. (2020). Glio-blastome Multiforme: A Bibliometric Analysis. World Neurosurg..

[28] Železnik D., Blažun Vošner H., Kokol P. (2017). A bibliometric analysis of the Journal of Advanced Nursing, 1976–2015. J. Adv. Nurs..

[29] Brandt J., Hadaya O., Schuster M., Rosen T., Sauer M.V., Ananth C.V. (2019). A Bibliometric Analysis of Top-Cited Journal Articles in Obstetrics and Gynecology. JAMA Netw. Open.

[30] Yang Q., Yang D., Li P., Liang S., Zhang Z. (2021). A Bibliometric and Visual Analysis of Global Community Resilience Research. Int. J. Environ. Res. Public Health.

[31] Chen W., Geng Y., Zhong S., Zhuang M., Pan H. (2020). A bibliometric analysis of ecosystem services evaluation from 1997 to 2016. Environ. Sci. Pollut. Res..

[32] Donthu N., Kumar S., Mukherjee D., Pandey N., Lim W.M. (2021). How to conduct a bibliometric analysis: An overview and guidelines. J. Bus. Res..

[33] Zhang Y., Lim D., Yao Y., Dong C., Feng Z. (2022). Global Research Trends in Radiotherapy for Gliomas: A Systematic Bibliometric Analysis. World Neurosurg..

[34] Yeung C.A., Chong L.-Y., Glenny A.-M. (2015). Fluoridated milk for preventing dental caries. Cochrane Database Syst. Rev..

[35] Falagas M.E., Pitsouni E.I., Malietzis G., Pappas G. (2008). Comparison of PubMed, Scopus, Web of Science, and Google Scholar: Strengths and weaknesses. FASEB J..

[36] Beovich B., Olaussen A., Williams B. (2021). A bibliometric analysis of paramedicine publications using the Scopus database: 2010–2019. Int. Emerg. Nurs..

[37] Keighobadi M., Nakhaei M., Sharifpour A., Khasseh A.A., Safanavaei S., Tabaripour R., Aliyali M., Abedi S., Mehravaran H., Banimostafavi E.S. (2021). A Bibliometric Analysis of Global Research on *Lophomonas* Spp. in Scopus (1933–2019). Infect. Disord.-Drug Targets.

[38] AlRyalat S.A.S., Malkawi L.W., Momani S.M. (2019). Comparing Bibliometric Analysis Using PubMed, Scopus, and Web of Science Databases. J. Vis. Exp..

[39] Van Eck N.J., Waltman L., Ding Y., Rousseau R., Wolfram D. (2014). Visualizing bibliometric networks. Measuring Scholarly Impact: Methods and Practice.

[40] Büttner W., Henschler D., Patz J. (1973). Kariesprophylaxe durch Fluorid-Einnahme. Fluorid in Blut und Speichel nach Zufuhr mit Trinkwasser und Tabletten [Caries prevention through fluoride intake. Fluoride in blood and saliva following administration in drinking water and tablets]. Dtsch. Med. Wochenschr..

[41] SCOPUS CiteScore Journal Metrics–FAQs. https://service.elsevier.com/app/answers/detail/a_id/30562/supporthub/scopus/session/.

[42] Stecksén-Blicks C., Sjöström I., Twetman S. (2009). Effect of long-term consumption of milk supplemented with probiotic lactobacilli and fluoride on dental caries and general health in preschool children: A cluster-randomized study. Caries Res..

[43] Ophaug R.H., Singer L., Harland B.F. (1985). Dietary fluoride intake of 6-month and 2-year-old children in four dietary regions of the United States. Am. J. Clin. Nutr..

[44] Stephen K.W., Boyle I.T., Campbell D., McNee S., Boyle P. (1984). Five-year double-blind fluoridated milk study in scotland. Community Dent. Oral Epidemiol..

[45] Wiegand A., Attin T. (2014). Randomised in situ trial on the effect of milk and CPP-ACP on dental erosion. J. Dent..

[46] Mariño R., Fajardo J., Morgan M. (2012). Cost-effectiveness models for dental caries prevention programmes among Chilean school-children. Community Dent. Health.

[47] Mariño R., Villa A., Guerrero S. (2001). A community trial of fluoridated powdered milk in Chile. Community Dent. Oral Epidemiol..

[48] Bánóczy J., Zimmermann P., Hadas E., Pinter A., Bruszt V. (1985). Effect of fluoridated milk on caries: 5 year results. J. R. Soc. Health.

[49] Pratten J., Bedi R., Wilson M. (2000). An In Vitro Study of the Effect of Fluoridated Milk on Oral Bacterial Biofilms. Appl. Environ. Microbiol..

[50] Bian J.Y., Wang W.H., Wang W.J., Rong W.S., Lo E.C.M. (2003). Effect of fluoridated milk on caries in primary teeth: 21-month results. Community Dent. Oral Epidemiol..

[51] Ketley C., Lennon M. (2001). Determination of Fluoride Intake from Urinary Fluoride Excretion Data in Children Drinking Fluoridated School Milk. Caries Res..

[52] Association of State and Territorial Dental Directors (ASTDD) Fluoride Supplement Policy Statement. https://www.astdd.org/docs/fluoride-supplement-policy-statement-january-28-2013.pdf.

[53] Zimmer S., Jahn K.-R., Barthel C.R. (2003). Recommendations for the use of fluoride in caries prevention. Oral Health Prev. Dent..

[54] Gov.UK Chapter 9: Fluoride. https://www.gov.uk/government/publications/delivering-better-oral-health-an-evidence-based-toolkit-for-prevention/chapter-9-fluoride.

[55] Fernández Fernández E., Martínez Hernández J.A., Martínez Suárez V., Moreno Villares J.M., Collado Yurrita L.R., Hernández Cabria M., Morán Rey F.J. (2014). Documento de consenso: Importancia nutricional y metabólica de la leche [Consensus document: Nutritional and metabolic importance of cow’s milk]. Nutr. Hosp..

[56] Protudjer J.L., Jansson S.A., Östblom E., Arnlind M.H., Bengtsson U., Dahlén S.E., Kallström-Bengtsson I., Marklund B., Middelveld R.J., Rentzos G. (2015). Health-related quality of life in children with objectively diagnosed staple food allergy assessed with a disease-specific questionnaire. Acta Paediatr..

[57] Africa.Com The Cash Cow of Africa–Dairy. https://www.africa.com/dairy-consumption-in-africa-part-1/.

[58] Food and Agriculture Organization of the United Nations Gateway to Dairy Production and Products. https://www.fao.org/dairy-production-products/processing/milk-preservation/en/.

[59] Lawson L.A. (2020). GHG emissions and fossil energy use as consequences of efforts of improving human well-being in Africa. J. Environ. Manag..

[60] Wen P., Chen M., Zhong Y., Dong Q., Wong H. (2022). Global Burden and Inequality of Dental Caries, 1990 to 2019. J. Dent. Res..

[61] Evans D. (2003). Hierarchy of evidence: A framework for ranking evidence evaluating healthcare interventions. J. Clin. Nurs..

[62] Salami A., Kanmodi K.K. (2021). Translational medical research in Nigeria: Challenges, prospects and recommendations for the future. Eur. J. Transl. Clin. Med..

